# Editorial: Towards a Meaningful Instrumental Music Education. Methods, Perspectives, and Challenges

**DOI:** 10.3389/fpsyg.2020.625994

**Published:** 2020-12-01

**Authors:** Andrea Schiavio, Luc Nijs, Dylan van der Schyff, Marja-Leena Juntunen

**Affiliations:** ^1^Centre for Systematic Musicology, University of Graz, Graz, Austria; ^2^IPEM, Department of Art, Music and Theatre Sciences, Ghent University, Ghent, Belgium; ^3^Melbourne Conservatorium of Music, The University of Melbourne, Melbourne, VIC, Australia; ^4^Faculty of Music Education, Folk music and Jazz, Sibelius Academy, University of the Arts Helsinki, Helsinki, Finland

**Keywords:** music teaching, instrumental music education, learning, meaning, music

Learning music is a complex, fascinating process that spans an impressive variety of meanings and experiences. The contributions that feature in this Research Topic bring together insights from a range of complementary perspectives to examine in detail how these layers of significance are part of, and shape, instrumental music education. To better capture the richness of such work, the present collection of articles is conceived around the following five themes: (i) body and action, (ii) technology, (iii) lived experience and meaning-making, (iv) pedagogical implications, and (v) beyond the musical instrument. It should be noted that many overlaps can be observed between the five topics, as insights developed in one specific area, as it often occurs in both the sciences and the humanities, may find a home (and be further developed) in other scholarly areas. For example, the analysis of lived experience has important implications for technology-enhanced learning and the modes of engagements one can develop with its tools and concepts; similarly, the study of action, movements, and gestures may stimulate novel pedagogical insights to transform existing learning paradigms and cultivate corporeal practices situated beyond music-only territories. Accordingly, while in what follows we examine each theme separately, we also highlight continuities and similarities emerging across manuscripts and topics.

## Body and Action

The main characteristics of what a meaningful instrumental music education involves can hardly be captured by rigid prescriptions or sets of rules determined a-priori. Instead, many scholars argue that aspects pertaining to body and action, which emerge and develop in the concrete, moment-to-moment dynamics of a music lesson, play a more fundamental role in driving meaningful teaching and learning (see e.g., Bowman, 2004). Accordingly, a close focus on their experiential and behavioral features can inspire important insights concerning how meaning is generated and transformed during musical practice. Three conceptual analysis articles published move from such a perspective, as they place a renewed emphasis on how teachers can meaningfully communicate with their students through gestures, touch, and other forms of engagement (Bremmer and Nijs; Simones), and how a phenomenological analysis of different bodily processes can illuminate on how musical skills are acquired contextually (Kim). In these contributions, theoretical backgrounds inspired by dynamical system theory and embodied cognitive science are used to frame research in various ways, disclosing potential real-life applications that inform theory and practice. For instance, researchers could develop a range of self-reflection tools that help describe in more detail the moment-to-moment dynamics of bodily activity, in turn supporting flexible ways of learning that take advantage of various resources a bodily approach can offer. This includes novel possibilities to engage with technological tools in meaningful ways, as we shall see next.

## Technology

As we are living in a highly technological age, digital technology imbues almost all aspects of life. This, accordingly, has also profound repercussions in the domain of music education. While digital technology is often seen as a force of change (see Savage, 2009), its acceptance and adoption are not self-evident. As Tuuri and Koskela state, a reason for this might involve considering technology as something unnatural, distant from how we live and develop experience contextually. However, moving from the idea that technology has been, and still is, an integral part of human evolution and flourishing, the authors propose to view technology as a co-constitutive aspect of making and understanding music. This insight is framed within a general perspective based on a post-phenomenological view on the human–technology relationship and on *4E cognition*, a school of thought in the cognitive science that conceives of mental life as an Embodied, Embedded, Extended, and Enactive phenomenon, and that places emphasis on how living systems and their environment meaningfully co-evolve (see Newen et al., 2018). It is argued that such a cross-disciplinary liaison may encourage pedagogical practices that are based on “possibilities, imagination, and relationality,” rather than on conformity to conventional ways of thinking. This requires an instructional design methodology that guides the integration of technology in music teaching and learning. According to Macrides and Angeli such a methodology is missing. To address this issue, their contribution aims to enrich the existing framework of *Technological Pedagogical Content Knowledge* (TPCK) by connecting affect and cognition to the affordances offered by technology within an educational design process. A final contribution that primarily focuses on technology is that of Addessi. Summarizing the state-of-the-art of the research project she coordinated (the MIROR project), the author describes the main applications of the MIROR platform (an educational device consisting of a set of software). The latter is described as a tool to stimulate and develop musical and motor creativity in children, particularly in areas such as dance or music composition. Taken together, these contributions emphasize the key role of a meaningful interaction with technology in pedagogical contexts, highlighting the importance of lived experience across different music-making activities.

## Lived Experience and Meaning-Making

Meaningfulness in music education, as in education in general, is largely related to how people make sense of their active participation and experiences, and how they find learning meaningful for themselves and for others. Silverman links meaningfulness to a 4E-inspired account of “sense-making” in/for instrumental music education. According to her, meaningfulness comes about as we engage with activities, objects, and persons in ways that “connect us both to ourselves and our worlds in significant ways.” Three contributions under this theme report on empirical studies. Schiavio et al. examined music teachers' experiences and perceptions of ensemble skills and learning skills when working with collaborative forms of music making. The ability to “listen and respond to others” emerged as the most important ensemble skill, whereas “time management,” “comparing yourself to the class,” and the “development of responsible ways of learning” were considered the main learning skills. The other two contributions report results from interpretative phenomenological analysis of performing musicians' lived experiences of Dalcroze practice—an approach to music education that facilitates musical exploration and enhances understandings of music-movement relationships through integration of group movement activities, ear-training, and creative engagement. The participating flute players found the approach useful in preparing repertoire for performance (Ridout and Habron). The instrumental ensemble performers had experienced a chain of influences including heightened awareness of music, time, space and energy as well as between the ensemble members, improvement of musicianship, decreased self-consciousness, and finally enjoyment that all enhanced learning (Wentink and Van der Merwe).

## Pedagogical Implications

The contributions of this collection, as we saw, can offer important insights into a variety of pedagogical settings ranging from the use of technological resources in class to the phenomenological analysis of lived experience in those who teach and learn music. An additional aspect this work can contribute to involves concrete implications for theory and practice across manifold educational contexts. For instance, while the article by Filippa et al. focuses on how infants and children imitate musical gestures, opening up fascinating pedagogical possibilities from early musical interventions, the article by Ford asks us to critically reflect upon the theme of interculturality in the music curriculum. This latter topic is closely related to that of collaborative learning, as explored by Johansen and Nielsen through the report of a workshop conducted by the two authors, where the experiences of students involved in peer-learning were assessed. Real-life examples of music pedagogy are also at the core of the empirically grounded contributions by Philippe et al., and Meissner and Timmers. The former article addresses the self-regulation processes involved in the preparation of a music exam; the latter, examines the role of dialogue teaching in learning to perform expressively. Both articles highlight a need to consider in more detail the experiential dynamics framing meaningful instrumental education. An important aspect of this enterprise, captured by the systematic review by Després and Dubé, involves listening more carefully to what students have to say in both in-school and out-of-school contexts, thereby providing a more nuanced perspective of their meaning-making possibilities in musical and non-musical contexts.

## Beyond the Musical Instrument

Another theme that emerges in this Research Topic concerns the uses of instrumental practice for facilitating learning and development in other domains, and for well-being more generally. Gutierrez discusses a novel approach for learning music theory that adapts the *Conduction* techniques introduced by jazz improviser Butch Morris. This approach involves a flexible lexicon of signs and gestures that are realized by the ensemble in real time. As Gutierrez explains, “In a theory-learning context, students bring their instruments to class, form an ensemble, and take turns using signs and gestures to conduct their peers, guided through processes aligned with learning objectives (e.g., harmonic minor scales, Neapolitan chords, or polytonality), as well as to more freely experiment with musical structure *in situ*, with minimal or no reliance upon notation.” This approach provides a welcome shift away from the abstract orientation that characterizes much music theory education, opening a more holistic, embodied, and interactive perspective. Gutierrez discusses how Conduction can be understood as a “4E music theory pedagogy” and draws inspiration from current work in embodied mathematics education. The effects of body-based forms of learning on mathematical skills are also discussed by Ribeiro and Santos. Their article reports on a longitudinal study that examines the impact of musical training on numerical cognition. In particular, they found that musical activities that involve melodic and rhythmic engagement correlate with improvements in the mathematical skills of participants who suffer from developmental dyscalculia. Discussing this finding from a 4E perspective, the authors suggest that musical training may afford a wider range of cognitive domains for learning and therefore offers “a useful tool for compensatory remediation” of learning disabilities. Along similar lines, MacRitchie et al. discuss a mixed-methods study that examines the impact music instrument training programs have for improving well-being in older adults. Specific attention is given to cognitive, motor, and social factors. This research provided mixed results, showing the motivational importance of contextual factors associated with the choice of repertoire and the level of cohesiveness that develops within a given group of participants. As the authors note, “the class itself may impact the cognitive gains that individual participants in that class experience.” Accordingly, the authors suggest that more research is needed to better understand the elements that encourage and impede positive class environments.

To conclude, we believe that all the contributions in this Research Topic address essential elements of instrumental music education; and that the findings, insights, and perspectives discussed in each paper could inspire new research and theory in this fast-evolving domain. We hope this collection will thus become a reference for continuing research in instrumental music education.

## Author Contributions

All authors listed have made a substantial, direct and intellectual contribution to the work, and approved it for publication.

## Conflict of Interest

The authors declare that the research was conducted in the absence of any commercial or financial relationships that could be construed as a potential conflict of interest.
